# Data on the cost effective surface sterilization method for *C.carandas* (L.) seeds and callus induction from aseptic seedling

**DOI:** 10.1016/j.dib.2016.04.047

**Published:** 2016-04-26

**Authors:** Bhushan S. Bhadane, Ravindra H. Patil

**Affiliations:** Plant Tissue Culture laboratory, Department of Microbiology and Biotechnology, R. C. Patel Arts, Commerce and Science College, Shirpur, 425 405, (MS) India

**Keywords:** *Carissa carandas*, Benzalkonium chloride, Plant growth regulators (PGRs), Callus induction

## Abstract

Surface sterilization of explant is an important and most sensitive step in plant tissue culture. Inappropriate concentrations of sterilants have lethal effect in cell division and it restricts growth and development of explant. Therefore, suitable concentration, combinations and duration of exposure of sterilant is essential to raise in vitro cultures successfully. This data demonstrates use of various sterilizing agents for aseptic plantlet germination from seed of *Carissa carandas* (Apocynaceae). The present dataset provides information in support of cost-effective explant sterilization potential of benzalkonium chloride containing commercial bleach (Lizol) and its comparison with traditionally used surface sterilants in plant tissue culture i.e. 0.1% HgCl_2_ alone and in combination with 70% alcohol. The data on callogenic response using MS medium supplemented with plant growth regulators is also shared.

**Specifications Table**

**Table:** 

Subject area	Biology
More specific ubject area	Explant sterilization and callus induction
Type of data	Text file, tables and figures
How data was acquired	Using plant tissue culture technique.
Data format	Analysed
Experimental factors	Explant (seeds of *C.carandas*) sterilization using chemical sterilants like benzalkonium chloride (0.1%) alone and in combination with 70% alcohol and 0.1% HgCl_2_ alone and its combination with 70% alcohol to get aseptic plantlet in MS (Murashigue and Skoog) medium.
Experimental features	The aseptic plantlets resulting after sterilization therefore used for callus induction study. The leaves of aseptic seedling treated using various concentrations and combinations of plant growth regulators used to analyze callogenic response.
Data source location	North Maharashtra, MS, India. (21.26°N and 75.11°E). Data analysis: Shirpur, MS, India
Data accessibility	The data is available with this article.

**Value of data**

• This data provides information about use of cheaper sterilant for effective explant sterilization and to reduce the cost of process.
• The data is valuable for the selection of appropriate sterilization method for recalcitrant seeds of some medicinal, horticultural plants as well as for other delicate and sensitive explants.
• The data provides information to induce friable and embryogenic callus using the proposed concentrations and combinations of PGRs.

## 1 Data

**Fig. 1:**
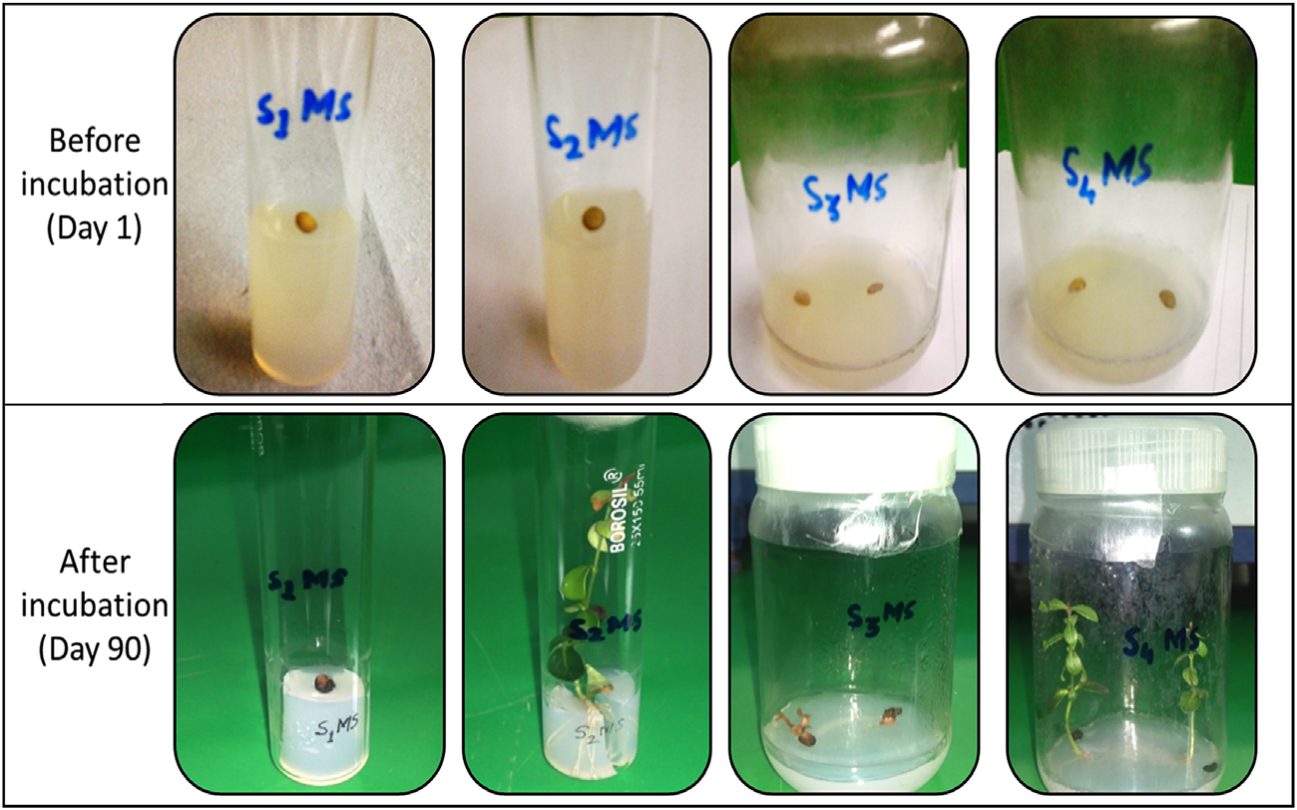
Effect of sterilants on *C. carandas* seeds.

**Fig. 2:**
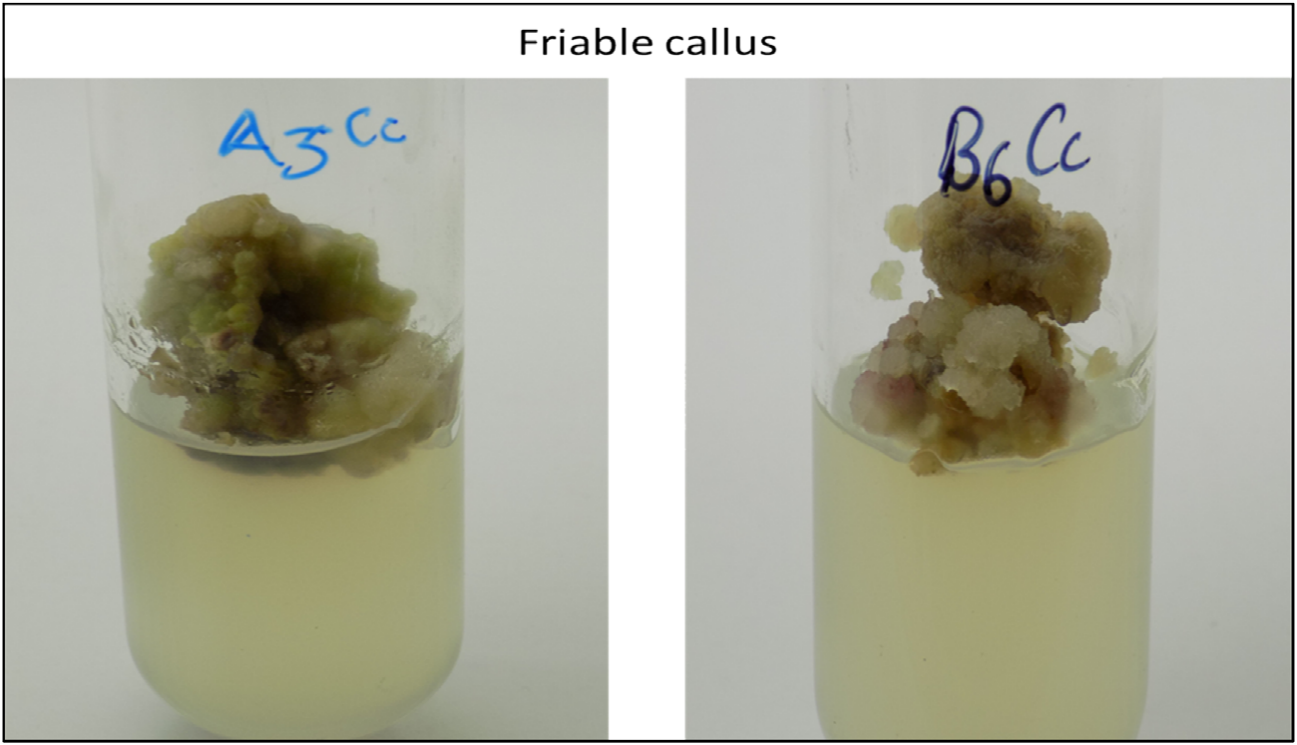
Induction of friable callus from aseptic seedlings using PGRs treatment.

**Fig. 3:**
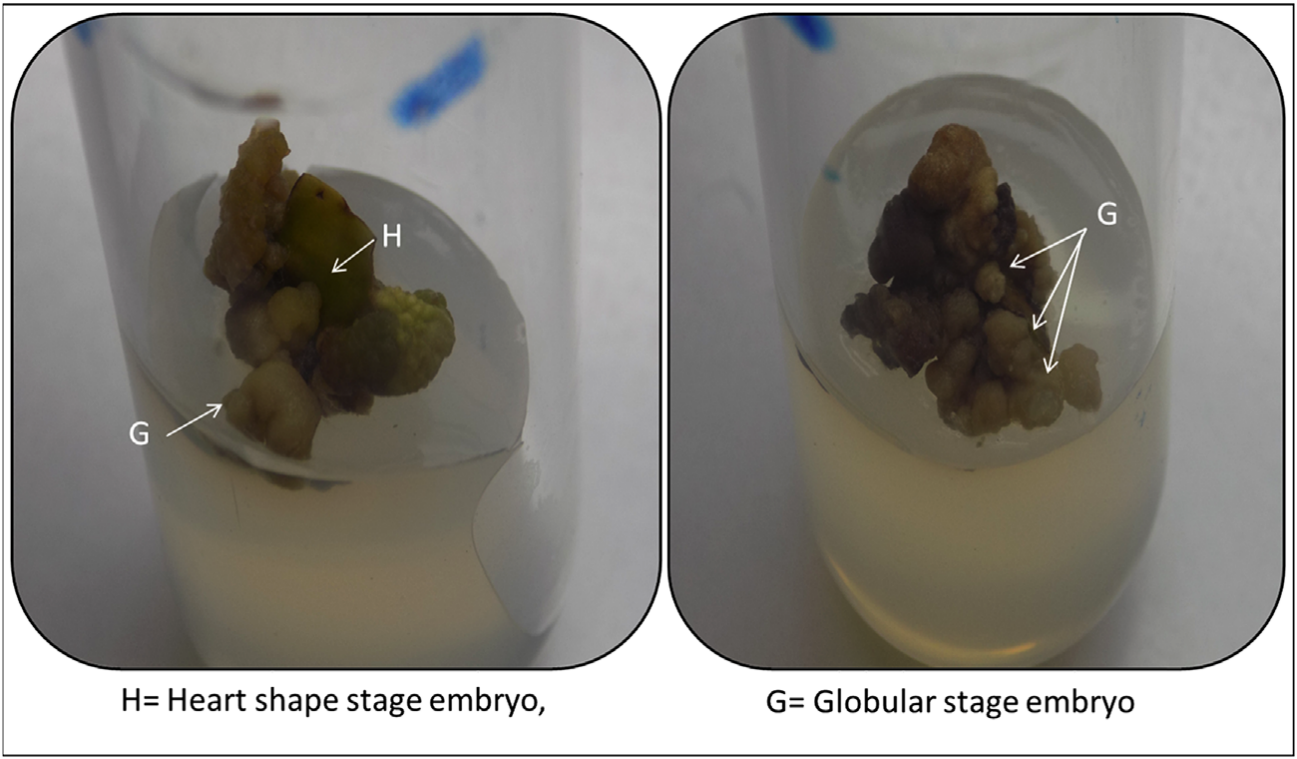
Embryogenic potential cells in A4 callus.

The data shared in this article is sterilization efficacy of benzalkonium chloride and callogenic response in presence of different PGRs. The significant difference in seed germination with the treatment using treatment code S1MS and S3MS even after 90 days of incubation indicates the adverse effects of HgCl_2_ on germination of *C. carandas* seeds (Fig. 1). This data also reveals that effect of PGRs treatment towards callogenic response and best response was recorded in media code A3 and B6 with friable callus (Fig. 2) while media code A4 gave embryogenic callus (Fig. 3).

## 2 Experimental design, materials and methods

Seeds of *C. carandas* are recalcitrant and having low viability, poor germination and they are most sensitive to chemical treatment [1], [2]. Hence, these seeds were used to investigate the effect of various sterilants to remove surface born microorganism without any adverse effect on seeds.

### 2.1 Treatment of *C. carandas* seeds with different sterilants to get aseptic seedlings

The sterilants used for explant sterilization with their concentration, combinations, and time of exposure are shown in Table 1.

### 2.2 Treatment of leaves of aseptic seedling with PGRs to induce callus

Leaves of aseptic seedlings were used as explant for callus induction. The PGRs like NAA, 2,4D (auxins) and BAP, Kinetin (cytokinins) were used at various concentrations and combinations for callus induction study. The callus induction protocol was grouped into two experimental units according to PGRs treatment. The outline of the protocol is shown in Table 2.

### 2.3 Data analysis

The data obtained were analysed using an analysis of variance (ANOVA) and means were performed by the Duncan׳s multiple range test.

## Figures and Tables

**Table 1:** Surface sterilization protocol for *C. carandas* seeds.

**Treatment code**	**Sterilization procedure**	**Drying and inoculation of explant**
**S**_**1**_**MS**	0.1% HgCl_2_+0.05% Tween 20 for 5 min followed by four times washing using sterile distilled water.	After surface sterilization, seeds were kept in a Petri dish containing sterile filter paper, allowed to air dry and then inoculated on MS medium without PGRs.
**S**_**2**_**MS**	0.1% benzalkonium chloride +0.05% Tween 20 for 5 min. followed by four times washing using sterile distilled water.
**S**_**3**_**MS**	70% ethanol for 30 s, washed thoroughly using sterile distilled water and then treated with 0.1% HgCl_2_+0.05% Tween 20 for 5 min followed by four times washing.
**S**_**4**_**MS**	70% ethanol for 30 s, washed thoroughly using sterile distilled water, and then treated with 0.1% benzalkonium chloride +0.05% Tween 20 for 5 min followed by four times washing.

**Table 2:** PGRs treatment groups for callus induction.

**Group I**			**Group II**		
**Media code**	**PGRs concentration (mg l**^**–1**^**)**		**Media code**	**PGRs concentration (mg l**^**–1**^**)**	
**NAA**	**BAP**	**2,4 D**	**Kin**

**A1Cc**	2	0.5	**B1Cc**	3	0.5
**A2Cc**	4	0.5	**B2Cc**	4	0.5
**A3Cc**	6	0.5	**B3Cc**	5	0.5
**A4Cc**	2	1	**B4Cc**	3	1
**A5Cc**	4	1	**B5Cc**	4	1
**A6Cc**	6	1	**B6Cc**	5	1
